# Trends in co-prescribed opioids and benzodiazepines, non-prescribed opioids and benzodiazepines, and schedule-I drugs in the United States, 2013 to 2019

**DOI:** 10.1016/j.pmedr.2023.102584

**Published:** 2023-12-29

**Authors:** Shaden A. Taha, Jordan R. Westra, Danyel H. Tacker, Mukaila A. Raji, Yong-Fang Kuo

**Affiliations:** a Center for Metabolic Health, University of Texas Medical Branch, Galveston, TX, USA; b Department of Preventive Medicine and Population Health, University of Texas Medical Branch, Galveston, TX, USA; c Office of Biostatistics, University of Texas Medical Branch, Galveston, TX, USA; d Department of Pathology, Anatomy, and Laboratory Medicine, West Virginia University, WV, USA; e Department of Internal Medicine, University of Texas Medical Branch, Galveston, TX, USA; f Sealy Center on Aging, University of Texas Medical Branch, Galveston, TX, USA

**Keywords:** Opioids, Benzodiazepines, Schedule I drugs, Drug abuse, Opioid misuse, Concomitant use, Trends, Joinpoint regression

## Abstract

Concurrent opioid and benzodiazepine users are at increased risk of overdose death, compared to opioid-only users. The objective of this study was to understand recent time trends in opioid and benzodiazepine concurrent use, misuse, and schedule-I drug use, and how these differ by age, sex and geographic region. Commercial, United States medical insurance claims data and urine drug test results from 2013 to 2019 were used to study the outcomes of concurrent use (n = 756,258), schedule-I drug use (n = 746,672) and prescription misuse (n = 452,523). Drug use outcomes were studied at quarterly time points for each year.

Data analysis included joinpoint regression models to estimate quarterly drug use rates, determined by positive urine tests for corresponding drug categories, and was conducted from November 2021 through January 2022. Concurrent use decreased from 19.3% to 9.8%, misuse generally decreased from 75.6% to 55.1%, and schedule-I use increased from 8.9% to 13.8%, from 2013 to 2019. Concurrent use decreased at greater rates after 2016, after the Centers for Disease Control and Food and Drug Administration guidelines against concurrent use were released, while schedule-I use increased, notably after the 2014 hydrocodone reschedule. This indicates a potential shift from prescription use to non-prescribed drug use, where most affected groups included males, younger individuals, and those residing in Northeastern regions.

Study results support public health initiatives focused on policy that increases access to multimodal pain management and substance use disorder management programs—critical steps in preventing patients from seeking non-prescribed drugs for self- medicating due to pain or addiction.

## Highlights

• Concurrent opioid and benzodiazepine use in United States adults decreased from 19.3% in 2013, to 9.8% in 2019.
• Concurrent use decreased at a greater rate after the 2016 Opioid Prescribing Guideline by the Centers for Disease Control and Prevention and warnings from the Food and Drug Administration were released.
• Schedule I drug use generally increased from 8.9% in 2013 to 13.8% through 2019, with a noticeable dip between Q3 and Q4 of 2013, after the Drug Enforcement Agency announced the rescheduling of hydrocodone.
• Prescription opioid or benzodiazepine misuse decreased from 75.6% in 2013 to 59.3% by the end of 2019.

## 1 Introduction

Opioid use is associated with risks of overdose and death; these risks are substantially increased in patients concurrently taking benzodiazepines and opioids, due to the exacerbated effect of respiratory suppression. (Dowell et al., 2016) Concurrent users are at least four times more likely to overdose or experience drug-related emergencies, compared to those taking an opioid alone. (Sun et al., 2017, Park et al., 2015).

To quell these effects, the Centers for Disease Control (CDC) recommends the avoidance of co-prescribing opioids and benzodiazepines, and the use of urine drug testing (UDT) to initially assess and continuously monitor patients taking either or both drugs. (Dowell et al., 2016) The Food and Drug Administration (FDA) also requires a “blackbox” warning to be included on all opioids and benzodiazepines, cautioning the risks of concurrent use. (FDA, 2016).

Trends in co-prescribing of opioids and benzodiazepines increased from early 2000 until 2012, after which two large-scale studies show a decreasing trend through 2018. (IQVIA Institute, 2022, Esechie et al., 2021) Because most studies on concurrent use have relied on prescription claims or dispensing data, it is less clear whether patient use reflects the same findings. Therefore, time trends in concurrent use positivity were examined using laboratory UDT results from a large insurance claims database, from 2013 to 2019.

National overdose death involving any opioids increased approximately 72% from 2016 to 2019, (Underlying Cause of Death, 2020) most of which have been attributed to non-prescribed opioids and illicit drugs. (Kuo et al., 2021, Scholl et al., 2018) Because it is common for patients to shift from prescription opioids to non-prescribed and illicit drug use, (Muhuri et al., 2013) trends in schedule I drug positivity (illicit drugs) and non-prescribed use of opioids or benzodiazepines were also assessed. We hypothesized that concurrent use shown in UDT results would decrease from 2013 to 2019. Schedule-I drug use and non-prescribed opioids or benzodiazepines were expected to increase as a result, to compensate for prescriptions that might have been restricted or discontinued.

## 2 Methods

### 2.1 Data

Optum’s Clinformatics Datamart (CDM) de-identified insurance claims data were used to pull medical claims and laboratory records by current procedural terminology (CPT), Healthcare Common Procedure Coding System (HCPCS), and logical observation identifiers, names, and codes (LOINC), for UDT associated with opioids, benzodiazepines or schedule I drugs (Appendix Table 1), from January 1, 2013 through December 31, 2019. This study was determined exempt by the Institutional Review Board at the University of Texas Medical Branch at Galveston. To assess yearly match rates between UDT and results, CPT/HCPCS codes were used to represent total UDT (Appendix Table 2), while lab results were counted using drug-specific LOINCs (Appendix Table 3). Details on match rates may be found in Appendix Table 4. Because match rates were unexpectedly low, and to avoid discarding 73.8% of UDT results that were unmatched or missing a standard CPT code, data from LOINC-pulled files were used for analysis, rather than matched data. Within each drug category, only LOINC records with interpretable alphabetic or numerical results were included, such as “pos”, or “<50”, respectively. Overall, the LOINCs with interpretable results in each drug category were 67%, 73%, and 70% for schedule 1, opioid, and benzodiazepine, respectively (Appendix Table 5).

### 2.2 Outcomes

Three non-overlapping outcomes of UDT-positivity were defined: concurrent use, schedule-I use, and non-prescribed opioid and/or benzodiazepine use (misuse). Appendix Table 5 shows how the study cohort for each outcome was generated.

Concurrent use was defined as the percent of individuals with both opioid- and benzodiazepine-positive UDT on the same day, within each quarter. In a sensitivity analysis, concurrent use was also defined as concurrent-positive UDT within 3 days. Similarly, schedule-I use rates were defined as the percent of individuals with positive UDT for any schedule-I use within each quarter. “Any” prescription misuse was defined as any UDT-positive rates for an opioid, benzodiazepine, or both, outside a defined compliance window each quarter, among patient records with ≥ 180 days continuous enrollment prior to the UDT. The opioid compliance window included the prescription fill date, plus days of supply, plus 7 days to account for the time opioids remain detectable in urine. The compliance period was calculated similarly for benzodiazepines, with an additional 30 days to account for long-acting benzodiazepines. In a sensitivity analysis, prescription misuse was counted only if all opioid and/or benzodiazepine use in a quarter was misuse. The misuse rate was the percent of individuals with any misuse among those with positive opioid or benzodiazepine UDT in a quarter. The cohort flowchart for each outcome may be found in Appendix Table 5.

### 2.3 Time

Time quarters were grouped as January-March (Q1), April-June (Q2), July-September (Q3), and October-December (Q4) in each year from 2013 to 2019.

### 2.4 Covariates

Age, sex and United States Census regions were used to stratify time trends. Region was categorized by the US Census Bureau, into the Northeast, South, Midwest and West regions. Age was categorized as < 50, 50–59, 60–69, and 70 years and above.

### 2.5 Analysis

Quarterly rates of UDT positivity were plotted for concurrent use, schedule-I use, and misuse by age, sex, and region. Joinpoint Regression models, with a maximum of 5 possible joinpoints, were conducted to evaluate any significant changes in time trends. A sequential application of the permutation test using 4500 possible randomly permuted data sets and a Bayesian information criterion were used to determine the optimal number of joinpoints. The slopes were estimated to represent change at a constant percentage every quarter linearly on a log scale. Joinpoint Regression Program 4.9.0.0 (National Cancer Institute), and SAS 9.4 (SAS Institute v. 9.4, Cary, NC) were used for all analyses.

## 3 Results

### 3.1 Concurrent use

**Fig. 1:**
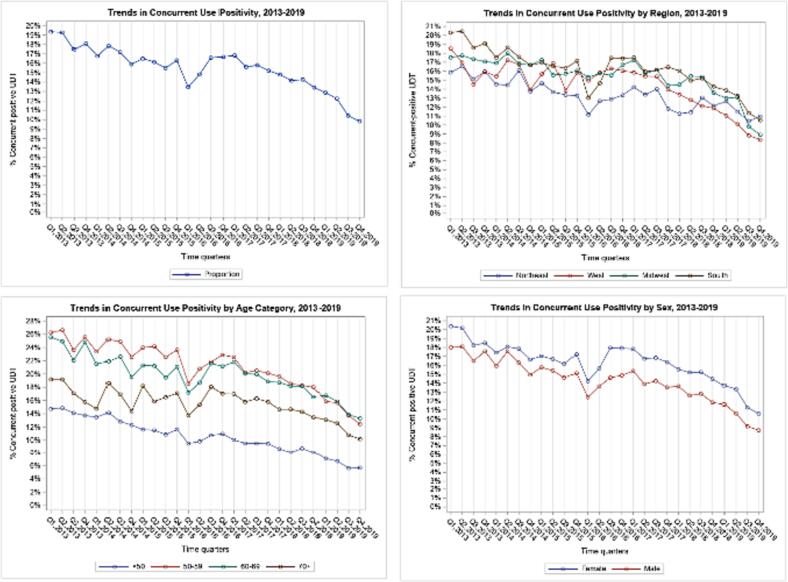
Panel of Graphs: Any Concurrent Opioid and Benzodiazepine Use, 2013–2019.

Fig. 1 shows trends in concurrent use in the total sample (n = 746,672 UDT) and stratified by age, sex and region. Among the total sample, concurrent use rates decreased from 19.3% in Q1 2013, to 9.8% by Q4 2019. Similar patterns were mostly observed by age, sex and region, where all groups’ concurrent use decreased with time. Notably, rates were higher in females than in males, lowest in age < 50, and highest in the 50–59 and 60–69 age groups. By region, concurrent use was highest in the South in 2013 (20.3%) and lowest in the Northeast (15.8%). However, by 2019, rates in the Northeast (10.9%) slightly surpassed the South (10.5%).

Table 1 presents log-scale slopes and detected joinpoints from joinpoint regression analysis of the studied outcomes. Concurrent-positive UDT showed a 2.3% quarterly decrease from Q1 2013 to Q1 2016, non-significant increase during 2016, 2.8% quarterly decrease from Q4 2016 to Q1 2019, and 9.5% quarterly decrease during 2019. In stratified analyses, joinpoints and slopes were slightly different in females compared to the entire study cohort. However, in males, only one significant joinpoint was found, showing a 1.35% quarterly decrease from Q1 2013 to Q3 2018, followed by a steeper quarterly decrease of 7.47% through Q4 2019.

**Table 1:** Joinpoints in trends and slopes of quarterly concurrent use, schedule I drug use, and any non-prescribed use, 2013–2019.

Concurrent Use^a^			Schedule I Drug Use			Non-Prescribed Use^b^		
Study period	Slope^c^	*P* value	Study period	Slope^c^	*P* value	Study period	Slope^c^	*P* value
**Total**			**Total**			**Total**		
Q1 2013 -Q1 2016	−2.3	<0.001	Q1 2013-Q1 2014	−8.29	0.061	Q1 2013-Q1 2016	−0.35	0.006
Q1 2016-Q4 2016	5.0	0.216	Q1 2014-Q2 2015	8.17	0.064	Q1 2016-Q4 2017	−1.57	< 0.001
Q4 2016-Q1 2019	−2.8	<0.001	Q2 2015-Q4 2019	1.58	< 0.001	Q4 2017-Q4 2018	−3.54	< 0.001
Q1 2019-Q4 2019	−9.5	<0.001				Q4 2018-Q4 2019	1.95	0.002
**Female**			**Female**			**Female**		
Q1 2013-Q1 2016	−2.18	< 0.001	Q1 2013-Q4 2013	−12.72	0.01	Q1 2013-Q1 2016	−0.33	0.014
Q1 2016-Q4 2016	6.06	0.128	Q4 2013-Q2 2015	7.86	0.003	Q1 2016-Q4 2017	−1.48	< 0.001
Q4 2016-Q2 2019	−3.05	< 0.001	Q2 2015-Q4 2019	2.11	< 0.001	Q4 2017-Q4 2018	−4.04	< 0.001
Q2 2019-Q4 2019	−12.36	0.004				Q4 2018-Q4 2019	1.78	0.005
**Male**			**Male**			**Male**		
Q1 2013-Q3 2018	−1.35	<0.001	Q1 2013-Q1 2014	−8.32	0.064	Q1 2013-Q2 2016	−0.39	0.001
Q3 2018-Q4 2019	−7.47	<0.001	Q1 2014-Q2 2015	7.83	0.084	Q2 2016-Q4 2018	−2.16	< 0.001
			Q2 2015-Q4 2019	1.32	< 0.001	Q4 2018-Q4 2019	1.74	0.007
**Age < 50**			**Age < 50**			**Age** < **50**		
Q1 2013-Q3 2018	−2.54	<0.001	Q1 2013-Q1 2014	−8.88	0.044	Q1 2013-Q1 2016	0.11	0.48
Q3 2018-Q4 2019	−8.17	<0.001	Q1 2014-Q4 2015	6.72	0.006	Q1 2016-Q4 2018	−1.73	< 0.001
			Q4 2015-Q4 2019	1.99	< 0.001	Q4 2018-Q4 2019	2.58	0.002
**Age 50**–**59**			**Age 50**–**59**			**Age 50**–**59**		
Q1 2013-Q3 2018	−1.42	<0.001	Q1 2013-Q1 2014	−8.49	0.058	Q1 2013-Q2 2017	−0.70	< 0.001
Q3 2018-Q4 2019	−7.85	<0.001	Q1 2014-Q3 2015	10.73	0.002	Q2 2017-Q4 2018	−3.37	< 0.001
			Q3 2015-Q4 2019	1.92	< 0.001	Q4 2018-Q4 2019	2.32	0.014
**Age 60**–**69**			**Age 60**–**69**			**Age 60**–**69**		
Q1 2013-Q1 2016	−2.35	<0.001	Q1 2013-Q4 2013	−9.36	0.325	Q1 2013-Q3 2017	−0.71	< 0.001
Q1 2016-Q1 2017	3.75	0.278	Q4 2013-Q1 2015	14.2	0.036	Q3 2017-Q4 2018	−3.96	< 0.001
Q1 2017-Q4 2019	−3.83	<0.001	Q1 2015-Q4 2019	3.19	< 0.001	Q4 2018-Q4 2019	2.03	0.001
**Age 70 and above**			**Age 70 and above**			**Age 70 and above**		
Q1 2013-Q4 2017	−0.40	0.268	Q1 2013-Q4 2019	4.15	< 0.001	Q1 2013-Q4 2015	−0.02	0.931
Q4 2017-Q4 2019	−4.80	<0.001				Q4 2015-Q4 2017	−1.51	<0.001
						Q4 2017-Q4 2018	−3.72	0.002
						Q4 2018-Q4 2019	0.84	0.252
**Northeast region**			**Northeast region**			**Northeast region**		
Q1 2013-Q4 2019	−1.24	<0.001	Q1 2013-Q1 2014	−8.98	0.075	Q1 2013-Q4 2014	−0.43	0.389
			Q1 2014-Q4 2019	1.82	<0.001	Q4 2014-Q3 2015	2.88	0.369
						Q3 2015-Q1 2019	−1.76	<0.001
						Q1 2019-Q4 2019	0.95	0.567
**West region**			**West region**			**West region**		
Q1 2013-Q3 2017	−0.32	0.237	Q1 2013-Q1 2014	−7.01	0.169	Q1 2013-Q1 2018	−1.06	<0.001
Q3 2017-Q4 2019	−0.614	<0.001	Q1 2014-Q4 2019	2.16	<0.001	Q1 2018-Q4 2018	−6.09	0.011
						Q4 2018-Q4 2019	1.68	0.035
**Midwest region**			**Midwest region**			**Midwest region**		
Q1 2013-Q3 2018	−0.72	<0.001	Q1 2013-Apr2014	−7.82	0.161	Q1 2013-Q3 2017	−0.4	<0.001
Q3 2018-Q2 2019	−6.06	0.301	Q2 2014-Q4 2019	2.59	<0.001	Q3 2017-Q3 2018	−3.55	0.006
Q2 2019-Q4 2019	−16.79	0.020				Q3 2018-Q4 2019	2.34	<0.001
**South region**			**South region**			**South region**		
Q1 2013-Q1 2016	−2.62	<0.001	Q1 2013-Q1 2014	−12.82	0.008	Q1 2013-Q2 2016	−0.25	0.070
Q1 2016-Q4 2016	5.71	0.242	Q1 2014-Q4 2015	8.18	<0.001	Q2 2016-Q4 2018	−2.31	<0.001
Q4 2016-Q1 2019	−2.39	<0.001	Q4 2015-Q4 2017	−0.94	0.268	Q4 2018-Q4 2019	1.92	0.014
Q1 2019-Q4 2019	−9.5	<0.001	Q4 2017-Q4 2019	2.68	<0.001			

Abbreviations: Q1: first quarter; Q2: second quarter; Q3: third quarter; Q4: fourth quarter

^a^ Sensitivity analysis where concurrent use was counted in opioid and benzodiazepine UDT up to three days apart showed three joinpoints at the same quarters found when defining concurrent use as UDT on the same day. The slopes were also very similar, with a 2.3% decrease quarterly from Q1 2013 to Q1 2016 (p < 0.001), 5.1% increase quarterly from Q1 2016 to Q4 2016 (p = 0.209), 2.8% quarterly decrease from Q4 2016 to Q1 2019 (p < 0.001) and 9.1% quarterly decrease from Q1 2019 to Q4 2019 (p < 0.001).

^b^ Sensitivity analysis of non-prescribed use showed 3 joinpoints, with slopes that decreased quarterly by 0.4% from Q1 2013 to Q1 2016 (p = 0.001), decreased 1.5% from Q1 2016 to Q4 2017 (p < 0.001), decreased 3.5% quarterly from Q4 2017 to Q4 2018 (p < 0.001), and increased 1.8% quarterly from Q4 2018 to Q4 2019 (p = 0.001).

^c^ Slope represents percent change of the quarterly rate of in UDT-positivity for each category of drug use linearly on a log scale.

Concurrent use decreased quarterly by 2.5% from Q1 2013 to Q3 2018, then by 8.2% through 2019 in the < 50 age group. Similar trends were observed in the 50–59 group. Among age 60–69, two significant joinpoints indicated a 2.3% quarterly decrease from Q1 2013 to Q1 2016, non-significant increase from Q1 2016 to Q1 2017, and a 3.8% quarterly decrease from Q1 2017 to Q4 2019. The smallest decrease was found in age > 70, with a 4.8% quarterly decrease from Q4 2017 through Q4 2019.

By region, concurrent use in the Northeast decreased 1.24% quarterly through the entire period. In the West, there was little change in concurrent use rates, decreasing 0.6% quarterly from Q3 2017 through Q4 2019. In the South, concurrent use decreased quarterly at greater rates with time (slope: −2.62% in Q1 2013-Q1 2016; slope: −2.39% from Q4 2016 to Q1 2019; slope: −9.5%, after Q1 2019); however, in the Midwest, there was a small decrease from Q1 2013 to Q2 2018 (slope: −0.7%), and a larger decrease from Q2 to Q4 2019 (slope: −16.7%).

### 3.2 Schedule I drug use

**Fig. 2:**
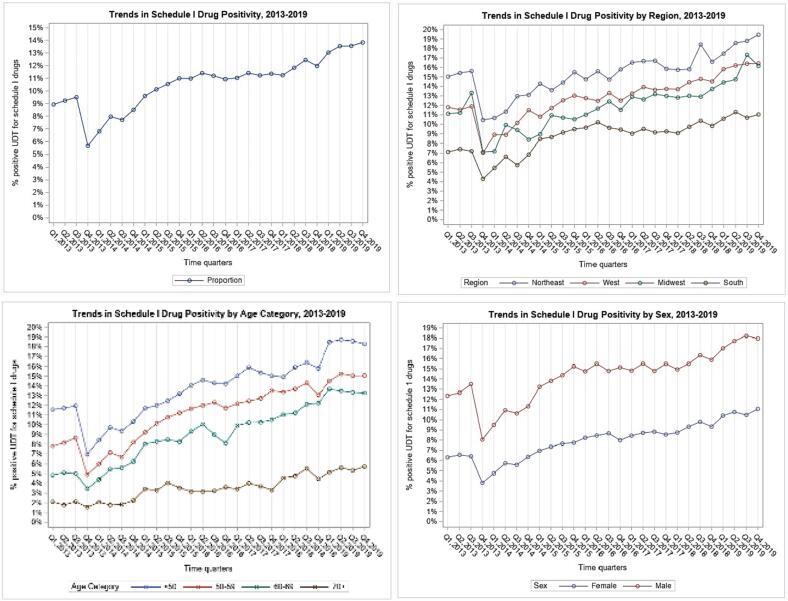
Panel of Graphs: Schedule I Drug Use, 2013–2019.

A total n = 756,258 UDT were included in the schedule I cohort. Schedule-I use generally increased from 8.9% in Q1 2013 to 13.8% in Q4 2019, with a noticeable dip between Q3 and Q4 of 2013, from 9.4% to 5.7% (Fig. 2). Higher schedule-I use was observed in males (12.4%, Q1 2013 to 17.9%, Q4 2019) than females (6.3%, Q1 2013 to 11.1%, Q4 2019). By region, lowest use was observed in the South (7%, Q1 2013 to 11.0%, Q4 2019), followed by the Midwest. The Northeast region had the highest rates, peaking at 19.4%, Q4 2019. By age, the rate of schedule-I use was highest among those < 50 (18.3%, Q4 2019). As age group increased, the magnitude of schedule-I drug use decreased; those ≥ 70 had the lowest use (5.7%, Q4 2019). Overall, schedule I drug use increased with time in all groups.

Joinpoint analysis of the total cohort detected two significant joinpoints, where there was an 8.3% quarterly decrease from Q1 2013 to Q1 2014, 8.2% increase from Q1 2014-Q2 2015, and a 1.58% increase from Q2 2015 to Q4 2019 (Table 1). Females had a higher quarterly decrease (12.7%) in illicit use from Q1 2013 to Q4 2013, which shifted to an increasing quarterly rate of 7.8% from Q 4 2013 to Q2 2015, followed by a slower quarterly increase of 2.1%, Q2 2015 to Q4 2019. Males showed a significant 1.3% quarterly increase from Q2 2015 to Q4 2019.

Age groups < 50, 50–59, and 60–69 each had two significant joinpoints; from Q1 2013 to Q1 2014, schedule-I use decreased 8.8% and 8.4% quarterly in age < 50 and 50–59, respectively, followed by a greater increase in age 50–59 group (slope:10.7%, Q1 2014 to Q3 2015), than age < 50 (slope: 6.7%, in 2014–2015), and the same 1.9% quarterly increase in both through Q4 2019. Age 60–69 showed similar results, although only increased by 3.2% quarterly in 2015–2019. Those ≥ 70 increased a steady 4.2% (p < 0.001) quarterly over the entire period.

By region, the Northeast, West and Midwest showed one significant joinpoint and similar trends. In 2014–2019, schedule-I use increased 1.8% and 2.1% quarterly in the Northeast and West, respectively. Similarly, there was a 2.5% quarterly increase in the Midwest, from Q2 2014 to Q4 2019. The South showed a large quarterly decrease of 12.8% from Q1 2013 to Q1 2014, 8.2% quarterly increase in 2014–2015, and slower increase of 2.7% quarterly from Q4 2017 to Q4 2019.

### 3.3 Non-prescribed use

**Fig. 3:**
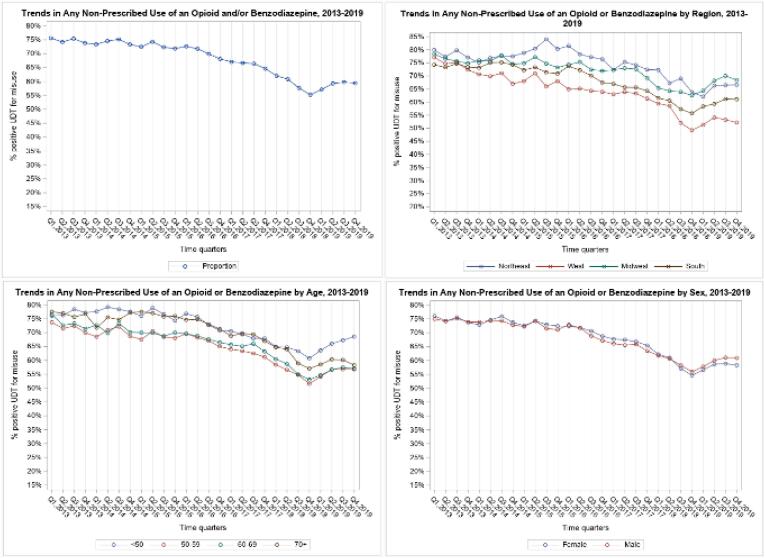
Panel of Graphs: Non-prescribed Use of Opioid and/or Benzodiazepines, 2013–2019.

Prescription misuse was assessed from n = 452,420 UDT. Overall, misuse decreased from 75.6%, Q1 2013 to 55.1%, Q4 2018, after which the rate increased again to 59.3% in Q4 2019 (Fig. 3). Similar patterns were observed by sex, although in Q1 2013 misuse was slightly higher in females (76.1%) than males (74.9%) and in Q4 2019, misuse was higher in males (60.9%) than females (58.3%). When stratified by age, misuse rates in 2013 were comparable (73.7–77.5%), decreased over time, and showed similar increases after Q4 2018 as in the total sample. By Q4 2019, ages 50–59, 60–69, and ≥ 70 had misuse rates of 56.7–58.3%, while age < 50 had a higher rate of 68.5%. In the West, Midwest and South, misuse generally decreased over the entire period, except for an increase from Q4 2018 – Q4 2019. In the Northeast, misuse appeared to fluctuate in early quarters then increase until Q3 2015, before decreasing again through Q4 2019. Differences by region were small in early quarters (74.3–79.9%) and varied more by Q4 2019 (52.2–68.3%).

In the total sample, three significant joinpoints were detected; misuse decreased 0.35% quarterly from Q1 2013 to Q1 2016, decreased 1.5% quarterly in 2016–2017, decreased 3.5% quarterly from Q4 2017 to Q4 2018, and increased 1.9% quarterly from Q4 2018 to Q4 2019 (Table 1). The same joinpoints and similar slopes were observed in misuse rates of females. Among males, only two joinpoints were found, with a similar pattern of slopes: small quarterly decrease, followed by a larger rate of decrease, and shift to increasing misuse after Q4 2018.

In age < 50, there was a 1.7% quarterly decrease in 2016–2018 and 2.5% increase from Q4 2018 to Q4 2019 in non-prescribed use, the highest slope among the age groups. Age 50–59 misuse decreased 0.7% quarterly Q1 2013 to Q2 2017, decreased 3.3% quarterly Q2 2017 to Q4 2018, and increased 2.3% quarterly Q4 2018 to Q4 2019. Age 60–69 had similar results to the 50–59 group. In age ≥ 70, significant slopes were found from Q4 2015 to Q4 2017 and from Q4 2017 to Q4 2018, with quarterly decreases of 1.5% and 3.7%, respectively.

In the Northeast region, between Q3 2015 to Q1 2019, misuse decreased 1.76% quarterly. In the West, misuse use decreased 1.1% quarterly from Q1 2013 to Q1 2018, decreased 6.1% quarterly during 2018, and increased 1.7% quarterly from Q4 2018 to Q4 2019. In the Midwest, misuse slightly decreased, by 0.4% quarterly Q1 2013 to Q3 2017, decreased 3.6% quarterly from Q3 2017 to Q3 2018, and increased 2.3% quarterly thereafter. Misuse in the South was similar to that of the Midwest, which decreased 2.3% quarterly from Q2 2016 to Q4 2018, and 2% through Q4 2019.

### 3.4 Sensitivity analyses

When defining concurrent use to include opioid and benzodiazepine UDT dates up to three days apart, similar concurrent use rates were observed. Overall concurrent use trends were similar to those observed in Fig. 1, decreasing from 19.3% in Q1 2013 to 9.8% in Q4 2019. Joinpoint analysis also showed very similar results.

## 4 Discussion

In this retrospective study of national laboratory data from commercial insurance claims 2013 to 2019, we observed decreasing time trends in concurrent opioid-benzodiazepine use and in non-prescribed prescription drug use accompanied by substantially increasing use of schedule-I drugs. Understanding trends in concurrent drug use has generally been limited to prescription claims studies, associated with providers’ behavior rather than patients. This study analyzed national laboratory results to gauge patient use and found that UDT trends are consistent with prior studies on co-prescribing, misuse and illicit drug use. (IQVIA Institute, 2022, Esechie et al., 2021, National Vital Statistics System, 2021).

The overall decreasing trend in concurrent drug use aligned with expectations from previous literature, (Esechie et al., 2021, Hwang et al., 2016, Koffel et al., 2020) which shows decreasing opioid and benzodiazepine co-prescriptions. Overall, concurrent use already decreased in 2013–2016, but decreased at a somewhat greater rate after the announcement and release of the 2016 CDC opioid-prescribing guideline, and decreased at a greater rate in 2019 after increased prevention measures were put in place by the Centers for Medicare and Medicaid Services (CMS). (Dowell et al., 2016, Announcement of Calendar Year (CY), 2019).

Among some groups, the change in concurrent use rates did not occur immediately after 2016, as seen in males, ages < 50, 50–59, ≥70, the West, and in the Midwest, which might indicate a delayed or lessened response to guidelines. Similarly, the linear decrease observed in the Northeast may reflect a lack of any response to the CDC guideline, although this region and the Midwest had smaller sample sizes, and patients of these regions receive UDT at lower rates than the South and West. (Taha et al., 2021) It is less clear why there was also a larger decline after 2018, however similar patterns were observed in national overdose deaths. (National Institute on Drug Abuse, 2021) Higher concurrent use in females was expected, as females are more likely to be prescribed opioids and benzodiazepines concurrently, (Sun et al., 2017) than males. People under 50 years had the lowest rates, while those 50–69 had the highest, consistent with a previous national study. (Vadiei and Bhattacharjee, 2020) Benzodiazepine use was also highest among 50–64 year-olds and may explain in part, the increased trends in this group. (Maust et al., 2019) The South had the highest concurrent use rates, which may be due to the increased prevalence of severe mental illness, (Lipari et al., 2013) UDT rates, and the large market share of CDM data in the South, and potentially indicate a lack of alternative treatments to co-prescribing in this region; (Sun et al., 2017, Taha et al., 2021, Vadiei and Bhattacharjee, 2020).

Schedule-I drug use increased across all ages, regions, and by sex, from Q2 2015 through Q4 2019. A sharp decline in schedule-I use was observed between the last two quarters of 2013, among females, those aged 18–59, and in the Northeast and South. One proposed explanation may be the effect of the Drug Enforcement Agency’s rescheduling of hydrocodone in 2014. Although this change occurred late in 2014, the FDA recommended the reschedule months prior, in December 2013. The drop in schedule-I use of the current study coincides with the greater rate of change in opioid-prescribing observed at a similar timepoint in a previous study. (Raji et al., 2018) Schedule-I use increased at a greater rate from 2014 to 2015 among females, those aged 18–69, and in the South, then increased at a slower rate. The dramatic increase from 2014 to 2015 may be due to less accessibility to hydrocodone after 2014.

Schedule-I use rates of 8.8–13.9% were generally consistent with recent CDC reports, which showed illicit drug use rates ranged from 8.1% to 23.9%, in adults aged 18 and above, by 2018. (National Center for Health Statistics, 2019) Males had higher rates of schedule-I drug use than females, which was expected. (Center for Behavioral Health Statistics and Quality, 2016) The Northeast had the highest schedule-I use of all regions, while the South had the lowest; (Illicit Drug Use, 2017) these regional differences have been observed previously, suggesting primarily illicit drugs use in the Northeast, and prescription opiate abuse in the South, shown by corresponding overdose deaths in these regions. Schedule-I use was lower in age ≥ 70 and increased as age decreased, which aligned with literature showing greater illicit use in younger individuals than older. (Nationl Survey on Drug Use and Health) Given that older individuals are up to 80% less likely to receive UDT than those under 50, it may explain why no change in illicit use was observed, and trends increased linearly in ages ≥ 70. (Taha et al., 2021).

We found 75.6% any misuse in Q1 2013 and 59.3% in Q4 2019. Previous studies have shown benzodiazepine misuse rates of 6.1–82.5%, and opioid misuse rates of 9.9–58%, though sample sizes and definitions of prescription misuse varied. (Blanco et al., 2018, Koyyalagunta et al., 2018, Hosain et al., 2019, Manthey et al., 2011) The somewhat higher rates of misuse found in this study may be due to the broader definition of non-prescribed use, which included those without a prescription, and those with prescriptions that may have been used in a manner not recommended by the provider. Additionally, misuse rates may be inflated due to selection bias of the misuse cohort; if providers sense a patient is at risk for misuse, there may be a differential in UDT requests, capturing more positive results among those more likely to misuse. (DiBenedetto et al., 2019, Hausmann et al., Jan 2013) UDT is also more common among patients that have indications for opioids or benzodiazepine use, such as those with various chronic pain indications and psychoses. (Taha et al., 2021).

The gradual decrease in misuse from 2013 to 2017 and the steeper decrease from 2017 to 2018 generally parallels the decreasing trend in co-prescribing opioids and benzodiazepines, and may indicate the shift to opioid and benzodiazepine alternatives, gabapentinoid and SSRI/SNRIs respectively, that provide a safer drug option, especially among the older population. (Esechie et al., 2021) However, the decrease was somewhat unexpected, given the recent increased mortality associated with prescription misuse. (Kuo et al., 2021) Beginning Q4 2018, non-prescribed use began to increase, which is consistent with the recent uptick in overdose deaths involving benzodiazepines and opioids between 2019 and 2020; (Chiappini et al., 2021) the abuse of prescribed and non-prescribed drugs has continued to increase in post-covid times due to increased “pharming”, “doctor-shopping”, drug shortages and healthcare strain in post-covid times, emphasizing the importance early drug abuse detection, prevention and pharmacovigilance Chiappini et al., 2022.

Considering the observed trends in the context of 1) findings on increased mortality from illicit use and prescription misuse, 2) few of such patients receive drug abuse treatment, and 3) the ineffectiveness of solely restricting opioid-prescribing, highlights the need for increased access to rehabilitation facilities for treatment. These findings also support the need for targeted public health initiatives, especially in males, younger individuals, and in Northeastern regions. The focus of public health efforts may require reorientation towards patient recovery rather than provider restriction.

## 5 Limitations

There are several limitations. First, insurance claims data limit generalizability to insured individuals, and therefore misses an important population affected by the opioid crisis—uninsured individuals—, which may underestimate drug use trends. Second, reliable race/ethnicity information is not available in CDM data and therefore could not be studied, though racial disparities in opioid compliance monitoring have been observed (Hausmann et al., 2013). Third, only independent laboratories were used, missing any tests that were done in a hospital setting. However, 116 labs were included; approximately 49% of results came from one large lab, 25% from another large lab, and 12% from a third lab. Fourth, some results were excluded for having uninterpretable values, which may have biased the sample, though it is uncertain how this could impact trends. However, the percent of annual UDT with interpretable results increased over time, indicating improvement of UDT results. Finally, match rates between procedure codes and results were unexpectedly low (Appendix Table 4). However, few UDT results included in the study were missing a CPT (<1%); instead, a nonstandard CPT code was often used, specific to benzodiazepines, opioids, and specific schedule-I drugs, though it’s unknown whether these codes link to standard CPT codes in a system outside of CDM data.

## 6 Conclusion

Concurrent opioid and benzodiazepine use decreased 2013–2019, and at a greater rate after the 2016 CDC guideline and FDA warnings against concurrent use, and in 2019 after CMS implemented increased safety measures against concurrent use. There was an increase in schedule-I use from 2013 to 2019 and a decline in prescription misuse, which began to increase after 2018. The continued increase in schedule-I drug use, while concurrent opioid and benzodiazepine use decreased, indicates a potential shift from prescribed to illicit use and emphasizes the need to support addiction recovery programs, and focus public health interventions on patient recovery and prevention.

## Funding

This work was supported by the National Institute on Drug Abuse [grant number R01-DA039192]. The funder had no role in the design, data collection, analysis or interpretation of the results.

This work was supported by grant R01-DA039192 from the National Institute on Drug Abuse. The funder had no role in the design and conduct of the study; collection, management, analysis, and interpretation of the data; preparation, review, or approval of the manuscript; and decision to submit the manuscript for publication.

## Declaration of competing interest

The authors declare that they have no known competing financial interests or personal relationships that could have appeared to influence the work reported in this paper.

## Figures and Tables

**Table A1:** List of drugs used to define opioid, benzodiazepine or schedule I drug UDT.

Opioids	fentanyl^a^, hydrocodone, hydromorphone, codeine, methadone, suboxone, propoxyphene, levorphanol, buprenorphine, morphine, oxycodone, meperidine, opioid panel
Benzodiazepines	flurazepam, oxazepam, clorazepate, chlordiazepoxide, alprazolam, lorazepam, clobazam, midazolam, temazepam, triazolam, diazepam, estazolam, benzodiazepine panel
Schedule I drugs^b^	heroin, cocaine, LSD, bath salts (cathinone/cathine), mescaline, psilocybin, MDMA, gamma-hydroxybutyric acid (GHB), marijuana (THC/cannabinoids), ecstasy, methaqualone, khat

a Because illicit fentanyl is indistinguishable from prescribed fentanyl by UDT, fentanyl was counted for the opioid drug category, and not in the schedule I category.

b As defined by the Food and Drug Administration.

**Table A2:** CPT and HCPCS codes used for all drug tests related to Schedule I drugs, opioids and benzodiazepines.

CPT/HCPCS^a^^,^^b^	Description	2013	2014	2015	2016	2017	2018	2019	Schedule I	Benzo	Opioid
G0659	definitive drug test	0	0	0	0	1	1	1	1	1	1
G0483	definitive drug test	0	0	0	1	1	1	1	1	1	1
G0482	definitive drug test	0	0	0	1	1	1	1	1	1	1
G0481	definitive drug test	0	0	0	1	1	1	1	1	1	1
G0480	definitive drug test	0	0	0	1	1	1	1	1	1	1
G0479	presumptive drug test	0	0	0	1	0	0	0	1	1	1
G0478	presumptive drug test	0	0	0	1	0	0	0	1	1	1
G0477	presumptive drug test	0	0	0	1	0	0	0	1	1	1
G6058	drug confirmation	0	0	1	0	0	0	0	1	1	1
G6056	opiates/metabolites	0	0	1	0	0	0	0	1	0	1
G6046	dihydromorphinone	0	0	1	0	0	0	0	1	0	1
G6045	dihydrocodeinone	0	0	1	0	0	0	0	1	0	1
G6044	cocaine	0	0	1	0	0	0	0	1	0	0
G6031	benzodiazepines	0	0	1	0	0	0	0	0	1	0
80,373	tramadol	0	0	1	1	1	1	1	0	0	1
83,925	opiates	1	1	0	0	0	0	0	1	1	1
82,649	dihydromorphinone	1	1	0	0	0	0	0	1	0	1
82,646	dihydrocodeinone	1	1	0	0	0	0	0	1	0	1
82,520	cocaine	1	1	0	0	0	0	0	1	0	0
G0434	presumptive drug test	1	1	1	0	0	0	0	1	1	1
G0431	presumptive drug test	1	1	1	0	0	0	0	1	1	1
83,992	phencyclidine	1	1	1	1	1	1	1	1	0	0
83,789	mass spectrometry drug test	1	1	1	1	1	1	1	1	1	1
80,365	oxycodone	0	0	1	1	1	1	1	0	0	1
80,364	opiates 5+	0	0	1	1	1	1	1	1	1	1
80,363	opiates 3–4	0	0	1	1	1	1	1	1	0	1
80,362	opiates 1–2	0	0	1	1	1	1	1	1	0	1
80,361	opiates 1+	0	0	1	1	1	1	1	1	0	1
80,360	methylphenidate	0	0	1	1	1	1	1	1	0	0
80,359	MDA/MDMA	0	0	1	1	1	1	1	1	0	0
80,358	methadone	0	0	1	1	1	1	1	1	0	1
80,356	heroin metabolite	0	0	1	1	1	1	1	1	0	1
80,354	fentanyl	0	0	1	1	1	1	1	1	0	1
80,353	cocaine	0	0	1	1	1	1	1	1	0	0
80,349	cannabinoids	0	0	1	1	1	1	1	1	0	0
80,348	buprenorphine	0	0	1	1	1	1	1	0	0	1
80,347	benzos 13+	0	0	1	1	1	1	1	0	1	0
80,346	benzos 1–12	0	0	1	1	1	1	1	0	1	0
80,307	presumptive drug test	0	0	0	0	1	1	1	1	1	1
80,306	presumptive drug test	0	0	0	0	1	1	1	1	1	1
80,305	presumptive drug test	0	0	0	0	1	1	1	1	1	1
80,304	presumptive drug test	0	0	1	1	0	0	0	1	1	1
80,303	presumptive drug test	0	0	1	1	0	0	0	1	1	1
80,302	presumptive drug test	0	0	1	1	0	0	0	1	1	1
80,301	presumptive drug test	0	0	1	1	0	0	0	1	1	1
80,300	presumptive drug test	0	0	1	1	0	0	0	1	1	1
80,154	benzodiazepines	1	1	0	0	0	0	0	0	1	0
80,102	presumptive drug test	1	1	0	0	0	0	0	1	1	1
80,101	presumptive drug test	1	1	0	0	0	0	0	1	1	1
80,100	presumptive drug test	1	1	0	0	0	0	0	1	1	1

^c^Indicators for schedule I drug, opioid or benzodiazepine were assigned to CPT/HCPCS codes and LOINCs. Distinct patient records that had both a valid CPT/HCPCS code and LOINC on the same drug test date in each respective category were considered a match.

a Some CPT/HCPCS codes were not specific to a drug class or metabolite, such as CPT code 80,307 for “presumptive drug testing”, which may include other drugs for testing.

b Due to the regular deletion or addition of billing codes annually, CPT and HCPCS codes differed by year, particularly for 2015.

**Table A3:** LOINC codes for Opioid, Benzodiazepine and Schedule I UDT.

Schedule I drugs	Benzodiazepines	Opioids
72795–8	59589–2	16334–5
67838–3	59590–0	18383–0
79237–4	94108–8	86604–6
72796–6	97159–8	49753–7
78858–8	94110–4	49751–1
79144–2	78758–0	58362–5
79236–6	28073–5	82371–6
87762–1	51776–3	77774–8
72797–4	61030–3	58361–7
73687–6	58365–8	89305–7
79238–2	94112–0	3508–9
72798–2	58364–1	16250–3
72793–3	19328–4	70206–8
73686–8	16348–5	3507–1
79242–4	61036–0	16197–6
72794–1	19326–8	13641–6
79232–5	19325–0	19411–8
79233–3	19330–0	19414–2
50594–1	19329–2	19413–4
3394–4	94115–3	51739–1
16226–3	58363–3	58391–4
70146–6	60677–2	89310–7
3393–6	49876–6	19449–8
14315–6	94105–4	19448–0
8192–7	86605–3	14066–5
8193–5	3313–4	16211–5
14314–9	59615–5	19446–4
43984–4	9351–8	19451–4
43985–1	16203–2	19450–6
19065–2	94116–1	51955–3
19358–1	78781–2	64131–6
19357–3	86224–3	51448–9
13479–1	42235–2	93465–3
19408–4	9428–4	93469–5
16632–2	20412–3	93461–2
19406–8	70140–9	74372–4
19405–0	52955–2	74810–3
19410–0	3390–2	93471–1
19409–2	16195–0	93467–9
86606–1	14316–4	11073–4
3398–5	70141–7	3637–6
20519–5	70142–5	78766–3
52953–7	19286–4	58381–5
3397–7	19064–5	11235–9
19360–7	19284–9	40839–3
19359–9	19283–1	59673–4
16448–3	3385–2	26867–2
19363–1	53745–6	78828–1
19362–3	90890–5	67822–7
53743–1	94117–9	79259–8
53747–2	3387–8	79260–6
86607–9	19282–3	58380–7
47400–7	19285–6	66129–8
42241–0	19280–7	58379–9
82723–8	19279–9	93470–3
5935–2	53736–5	11075–9
5939–4	41467–2	58383–1
19361–5	3459–5	43199–9
5937–8	20522–9	43200–5
50592–5	59879–7	95797–7
97154–9	3458–7	77775–5
80144–9	19386–2	58382–3
59295–6	19385–4	65808–8
28065–1	19388–8	81754–4
28044–6	19387–0	89306–5
43198–1	66128–0	93466–1
68440–7	19402–7	3681–4
29868–7	16229–7	78769–7
57746–0	50843–2	16252–9
43197–3	19245–0	12308–3
44358–0	16204–0	19483–7
47542–6	19399–5	19482–9
5608–5	19404–3	78830–7
16492–1	19403–5	74760–0
12295–2	86225–0	19485–2
10975–1	12314–1	19484–5
19593–3	3386–0	51738–3
55520–1	3388–6	58393–0
70197–9	20531–0	61422–2
10976–9	20534–4	61421–4
19322–7	16744–5	89304–0
19321–9	3551–9	78868–7
19324–3	16227–1	61420–6
19323–5	3550–1	9835–0
58384–9	18388–9	16998–7
51740–9	19443–1	9834–3
47004–7	19445–6	18473–9
20514–6	19444–9	19486–0
19592–5	64130–8	19488–6
19591–7	21241–5	19487–8
19596–6	3655–8	51737–5
19595–8	16231–3	46971–8
21050–0	3654–1	58394–8
27073–6	19475–3	89309–9
19594–1	19474–6	27076–9
14845–2	19477–9	3713–5
3547–7	19476–1	3712–7
16755–1	11238–3	20540–1
3546–9	20500–5	3711–9
16198–4	19490–2	16213–1
19439–9	19489–4	19516–4
19442–3	19492–8	19519–8
19441–5	19491–0	19518–0
86194–8	12602–9	3748–1
86195–5	20532–8	3747–3
86192–2	19494–4	16253–7
86196–3	19493–6	3746–5
79234–1	19496–9	16207–3
79235–8	19495–1	19532–1
3440–5	12432–1	58953–1
3439–7	20501–3	19535–4
3438–9	20533–6	19534–7
78817–4	19497–7	58386–4
79141–8	20535–1	72384–1
79243–2	20536–9	58385–6
79244–0	3725–9	3869–5
79246–5	17088–6	58389–8
79241–6	47115–1	27920–8
86193–0	18187–5	58388–0
45185–6	16205–7	95798–5
78757–2	19520–6	77777–1
3733–3	19523–0	58387–2
5679–6	19522–2	12369–5
20542–7	94109–6	27993–5
33350–0	58366–6	29656–6
3732–5	19588–3	45272–2
16214–9	16233–9	50542–0
19528–9	19586–7	58429–2
19531–3	19585–9	41858–2
19530–5	19590–9	58428–4
27085–0	19589–1	93495–0
45143–5	86231–8	77754–0
61048–5	19617–0	77752–4
59844–1	16230–5	58390–6
77772–2	61040–2	3775–4
64127–4	19615–4	3774–7
54943–6	19614–7	78770–5
91037–2	19619–6	16246–1
18355–8	19618–8	70147–4
20545–0	64129–0	52958–6
42253–5	27036–3	3773–9
19566–9	33301–3	16199–2
19565–1	15372–6	19550–3
20546–8	16228–9	70148–2
19567–7	70213–4	70149–0
64138–1	3861–2	78857–0
19570–1	16202–4	19553–7
18358–2	19624–6	19552–9
14267–9	19627–9	33527–3
19569–3	19626–1	41466–4
19568–5	58367–4	42251–9
19572–7	3887–7	3832–3
19571–9	16201–6	3831–5
73971–4	70214–2	78768–9
44424–0	12361–2	16251–1
9726–1	19639–4	70210–0
20543–5	19638–6	3830–7
12296–0	19641–0	16196–8
19543–8	19640–2	13648–1
19542–0	94107–0	19597–4
19545–3	58368–2	78861–2
19544–6	12382–8	19600–6
3787–9	20559–1	19599–0
16244–6	61060–0	3829–9
59928–2	16206–5	20550–0
3786–1	19698–0	3828–1
18389–7	19701–2	19602–2
19558–6	19700–4	19601–4
19560–2	94106–2	19604–8
19559–4	58369–0	19603–0
42252–7	4070–9	58392–2
93472–9	16232–1	89308–1
93468–7	14192–9	5707–5
93460–4	16224–8	5706–7
93457–0	19714–5	8220–6
93458–8	19717–8	17384–9
93473–7	19716–0	70150–8
93455–4	12374–5	52952–9
93456–2	59635–3	3879–4
93462–0	59888–8	18390–5
93451–3	52959–4	8221–4
93463–8	94114–6	8222–2
93464–6	3598–0	19295–5
93474–5	59672–6	21431–2
93459–6	97160–6	70151–6
93453–9	59938–1	19302–9
93454–7	94113–8	19138–7
93452–1	12311–7	19300–3
3937–0	17479–7	19299–7
16254–5	19679–0	19298–9
72825–3	19678–2	19301–1
52951–1	19681–6	19297–1
3936–2	19680–8	19296–3
14311–5		41465–6
18392–1		90894–7
8237–0		89302–4
8238–8		86609–5
19659–2		11246–6
14310–7		78767–1
19661–8		16249–5
19660–0		70215–9
58397–1		10998–3
19689–9		19643–6
6930–2		19642–8
19688–1		78873–7
17504–2		94304–3
19687–3		19645–1
19686–5		19644–4
19691–5		51954–6
19690–7		46973–4
80113–4		58395–5
78754–9		61197–0
21044–3		58430–0
88022–9		60276–3
87495–8		94305–0
87494–1		19648–5
87493–3		78765–5
87492–5		17395–5
87491–7		11247–4
72817–0		18325–1
72463–3		19646–9
78783–8		78874–5
72818–8		19650–1
78813–3		19649–3
78814–1		51736–7
95075–8		46975–9
42860–7		58396–3
20413–1		89301–6
70143–3		3919–8
52954–5		3918–0
3427–2		59951–4
19289–8		3917–2
18282–4		16219–8
70144–1		53781–1
70145–8		22065–7
26760–9		3544–4
19294–8		19635–2
19073–6		17377–3
19292–2		3871–1
19291–4		17376–5
19290–6		19632–9
19293–0		19637–8
67126–3		19636–0
19288–0		58399–7
19287–2		3545–1
26747–6		16242–0
3436–3		59960–5
20521–1		19141–1
3435–5		16200–8
19382–1		19429–0
19381–3		19436–5
19384–7		19435–7
19383–9		19434–0
19055–3		19433–2
72776–8		16749–4
72777–6		19438–1
8173–7		19437–3
20524–5		19432–4
3529–5		19431–6
19703–8		58398–9
19702–0		3416–5
19705–3		3415–7
19704–6		49752–9
73914–4		3414–0
72459–1		16208–1
78836–4		93494–3
72808–9		16496–2
72809–7		69033–9
72782–6		77764–9
72461–7		77787–0
78837–2		58360–9
72783–4		38373–7
72780–0		58359–1
72781–8		91027–3
73918–5		9396–3
72460–9		18435–8
72464–1		86610–3
78838–0		65807–0
72778–4		58402–9
72465–8		58401–1
78839–8		72485–6
72779–2		77879–5
72875–8		79240–8
72874–1		75362–4
72806–3		89300–8
72807–1		61429–7
77771–4		92904–2
78840–6		86454–6
78841–4		87760–5
72474–0		18338–4
78842–2		92639–4
78843–0		86453–8
72462–5		95799–3
78844–8		17719–6
78845–5		20561–7
78846–3		43219–5
73917–7		17718–8
72802–2		19710–3
72467–4		19713–7
72803–0		19712–9
72466–6		58404–5
78850–5		
72473–2		
72470–8		
78878–6		
72469–0		
78879–4		
78880–2		
72468–2		
78881–0		
72478–1		
3530–3		
14313–1		
3426–4		
17872–3		
19416–7		
8174–5		
8175–2		
19415–9		
21557–4		
21556–6		
14312–3		
19418–3		
19417–5		
13478–3		
72471–6		
72472–4		
88023–7		
82849–1		
82848–3		
82861–6		
82862–4		
90746–9		
87485–9		
87490–9		
87489–1		
87488–3		
87484–2		
82878–0		
82879–8		
72804–8		
78847–1		
72805–5		
78848–9		
78849–7		
87487–5		
90747–7		
87486–7		

**Table A4:** CPT/HCPCS and LOINC Urine Drug Test Match rates.

		2013	2014	2015	2016	2017	2018	2019	Total
(1) Denominator (UDT CPTs from medical claims)	(a) Total^a^	2,498,414	3,040,025	4,016,981	2,407,000	2,487,679	2,525,005	2,567,906	19,543,010
	(b) Independent Labs^b^	1,754,510	2,055,384	2,644,958	1,043,290	1,061,640	1,088,403	933,146	10,581,331
	(c) Total consolidated^c^	1,336,615	1,487,391	2,163,096	978,327	1,004,843	1,033,852	904,735	8,908,859
(2) Numerator (UDT LOINCs from lab claims)	(a) Total LOINC records	3,063,782	2,768,202	2,553,245	3,417,170	4,637,687	6,326,616	5,590,132	28,356,834
	(b) Total consolidated^d^	406,419	417,475	512,677	721,650	909,715	1,099,469	1,107,920	5,175,325
	(c) Matched^e^	261,043	223,509	216,599	135,087	172,396	198,332	148,502	1,355,468
	(d) Unmatched^f^	145,376	193,966	296,078	586,563	737,319	901,137	959,418	3,819,857
Match Rate	2c/1c	19.53%	15.03%	10.01%	13.81%	17.16%	19.18%	16.41%	15.21%

^a^ All records that included CPT/HCPC codes related to UDT for schedule I drugs, opioids or benzodiazepines.

^b^ CDM only includes lab results from independent laboratories, therefore tests (CPT/ HCPCS) codes were limited to those from independent labs.

^c^ Independent lab records were consolidated by person, date and CPT/HCPCS code.

^d^ Because a single CPT could be associated with many LOINCs (due to drug panels) the lab records were consolidated based on person, date, and test type (opioid, benzo, schedule 1) prior to matching.

^e^ Indicators for schedule I drug, opioid or benzodiazepine were assigned to CPT/HCPCS codes and LOINCs. Distinct patient records that had both a valid CPT/HCPCS code and LOINC on the same drug test date in each respective category were considered a match.

^f^ Although few LOINCs had a missing CPT (<1%) in each drug category, a large portion of LOINCs included in the cohorts (54.6% of schedule I UDT, 64.9% of benzodiazepine UDT, 67.5% of opioid UDT) were associated with unexpected CPT codes. These included invalid CPT codes such as ‘99999′ or ‘1111′, valid CPT codes that were broad (82540 “chemistry procedure”), or non-standard CPT values that may be laboratory-specific, such as “OPI_2” for opioids, or “BZD_2” for benzodiazepines, which led to a non-match.

**Table A5:** Cohort flowchart.

	2013	2014	2015	2016	2017	2018	2019	Total
Total UDT LOINC records	3,063,782	2,768,202	2,553,245	3,417,170	4,637,687	6,326,616	5,590,132	28,356,834
Schedule 1 UDT Records	619,922	534,612	485,253	719,004	943,440	1,259,896	1,202,946	5,765,073
UDT records with results^a^	283,165	264,101	290,914	515,136	737,175	926,996	866,352	3,883,839
UDT consolidated^b^	83,622	83,631	90,488	137,661	160,656	194,529	195,262	945,849
Selected UDT^c^	62,754	60,459	66,431	106,340	135,171	162,596	162,507	756,258
Opioid UDT total LOINC	1,680,332	1,533,339	1,447,865	1,937,942	2,698,663	3,581,014	3,082,370	15,961,525
UDT records with results^a^	932,923	857,050	762,666	1,414,382	2,215,909	2,865,811	2,655,119	11,703,860
UDT consolidated^b^	87,286	88,414	104,940	150,897	183,403	223,489	220,270	1,058,699
Benzo UDT total LOINC	763,528	700,251	620,127	760,224	995,584	1,485,706	1,304,816	6,630,236
UDT records with results^a^	403,919	379,144	307,581	515,683	773,972	1,156,376	1,096,984	4,633,659
UDT consolidated^b^	81,560	81,242	89,981	134,919	162,305	199,097	202,266	951,370
Concurrent UDT (same day)								
UDT consolidated^b^	81,101	80,705	88,973	133,197	160,257	195,959	198,935	939,127
Select UDT^c^	60,992	58,778	65,712	103,056	133,984	161,866	162,284	746,672
Concurrent Use Positive	11,320	9,919	10,569	16,004	21,244	22,942	18,537	110,535
Concurrent UDT (3 days)								
UDT consolidated^b^	93,027	94,943	102,256	147,029	173,709	214,257	219,614	1,044,835
Select UDT^c^	60,992	58,781	65,717	103,068	134,079	161,997	162,441	747,075
Concurrent Use Positive	11,325	9,924	10,575	16,019	21,307	23,020	18,596	110,766
Non-prescribed Use								
Positive opioid UDT	45,468	42,619	52,656	80,616	109,377	133,090	123,376	587,202
180-day eligibility prior to opioid UDT	38,811	36,170	43,194	65,186	91,065	113,219	105,118	492,763
Positive benzo UDT	18,025	16,653	17,914	26,267	33,543	38,563	33,809	184,774
180-day eligibility prior to benzo UDT	15,353	14,034	14,422	21,015	27,704	32,760	28,676	153,964
Select UDT^d^	35,027	32,395	38,923	59,280	84,373	104,924	97,601	452,523
Any misuses	26,573	24,330	28,580	42,000	56,377	62,688	58,068	298,616
Benzodiazepine misuse only	5,425	5,358	5,813	8,818	10,681	11,979	9,262	57,336
Opioid misuse only	16,184	14,619	18,310	26,942	37,992	43,794	43,349	201,190
Both misused	4,964	4,353	4,457	6,240	7,704	6,915	5,457	40,090
No misuse	8,454	8,065	10,343	17,280	27,996	42,236	39,533	153,907
Select UDT^e^	35,015	32,380	38,910	59,273	84,351	104,902	97,589	452,420
All misuse	26,169	23,980	28,265	41,576	55,787	61,771	57,388	294,936
Benzodiazepine misuse only	5,484	5,394	5,877	8,863	10,751	11,949	9,248	57,566
Opioid misuse only	15,880	14,341	18,045	26,585	37,505	43,063	42,765	198,184
Both misused	4,805	4,245	4,343	6,128	7,531	6,759	5,375	33,811
No misuse	8,846	8,400	10,645	17,697	28,564	43,131	40,201	157,484

a Of the 20,221,358 UDT records with interpretable results, 3,883,839 (19.2%) were schedule I UDT, 4,633,659 (22.9%) were benzodiazepine UDT, and 11,703,860 (57.9%) were opioid UDT.

b Tests were consolidated by UDT date; whether a patient received a panel for multiple drugs within a drug use category (schedule I, benzo, opioid), or a single test for any of these, it was counted once. The median (interquartile range) for number of UDT per person, per date increased from 3(2,4) in 2013 to 4(2,6) in 2019 in the schedule I category, decreased from 11 (4,16) in 2013 to 8(3,24) in 2019 among opioid UDT, and decreased from 6(1,8) to 1(1,12) in benzodiazepine UDT. Decreases in median tests among opioids and benzodiazepines occurred in 2015–2019, where LOINCs for panels such as “benzodiazepine panel” (LOINC 3390–2), or “opiates panel” (LOINC 3879–4) were more frequently use, than individual LOINCs for multiple drugs.

c Any positive UDT test in a quarter was selected. For those without positive results, the first UDT per person, per quarter was selected.

d Any misuse for an opioid or benzodiazepine in a quarter was selected, which may overestimate misuse. For those without any misuse, the first positive UDT per person, per quarter was selected. In the case of multiple tests where some indicated misuse and others indicated compliance, any misuse in a quarter was chosen which overestimated the rate of misuse.

e Misuse was counted if all opioid and/or benzodiazepine use in a quarter was misuse, which underestimated the rate of misuse.

## Data Availability

The authors do not have permission to share data.
